# Cell-free plasma telomere length correlated with the risk of cardiovascular events using machine learning classifiers

**DOI:** 10.1038/s41598-024-76686-2

**Published:** 2024-12-05

**Authors:** Mengjun Dai, Kangbo Li, Mesud Sacirovic, Claudia Zemmrich, Oliver Ritter, Peter Bramlage, Anja Bondke Persson, Eva Buschmann, Ivo Buschmann, Philipp Hillmeister

**Affiliations:** 1 Department for Angiology, Center for Internal Medicine I, Deutsches Angiologie Zentrum Brandenburg – Berlin (DAZB), University Clinic Brandenburg, Brandenburg Medical School Theodor Fontane, Brandenburg an der Havel, Germany; 2 Charité – Universitätsmedizin Berlin, Corporate Member of Freie Universität Berlin and Humboldt- Universität zu Berlin, Berlin, Germany; 3 Department for Cardiology, Center for Internal Medicine I, Brandenburg Medical School Theodor Fontane, University Clinic Brandenburg, Brandenburg an der Havel, Germany; 4 Faculty of Health Sciences Brandenburg, Brandenburg Medical School Theodor Fontane, Joint Faculty of the Brandenburg University of Technology Cottbus – Senftenberg, University of Potsdam, Brandenburg an der Havel, Germany; 5 Institute for Pharmacology and Preventive Medicine, Cloppenburg, Germany; 6 Department of Cardiology, University Clinic Graz, Graz, Austria

**Keywords:** Coronary artery disease, Machine learning, Heart failure, SHAP, Telomere length, Diagnostic markers, Predictive markers

## Abstract

This retrospective study explored the association between circulating cell-free plasma telomere length (cf-TL) and coronary artery disease (CAD) and heart failure (HF). Data from 518 participants were collected, including clinical and laboratory data. cf-TL was measured in plasma samples and machine learning (ML) classification models were developed to differentiate between CAD, HF and control conditions. Our results showed that cf-TL was significantly prolonged in HF patients compared to controls, but no significant difference was observed between CAD patients and controls. Additionally, cf-TL was significantly correlated with nitric oxide metabolites (NOx) and flow-mediated dilation (FMD), suggesting a potential link with endothelial function. To avoid data leakage and ensure the model captured only relationships relevant to the research question, we utilized a temporal data split, holding out the last year’s data for testing (n = 81) and using the remaining data for training (n = 324) and validation (n = 109). The ML models using four variables achieved an area under the curve (AUC) of 0.795 in the validation dataset and 0.717 in the test dataset for CAD classification, and 0.829 in the validation dataset and 0.806 in the test dataset for HF classification. SHAP analysis revealed that cf-TL had minimal impact on the predictions of the CAD model, as indicated by consistently low SHAP values, whereas in the HF model, cf-TL exhibited a broader range of SHAP values, indicating a greater contribution to the model’s classification. These findings suggest that cf-TL may play a more prominent role in HF pathophysiology and could serve as a valuable biomarker for predicting HF risk. Further studies are warranted to explore cf-TL’s diagnostic and prognostic potential across different cardiovascular diseases.

**Supplementary Information:**

The online version contains supplementary material available at 10.1038/s41598-024-76686-2.

## Introduction

Cardiovascular diseases (CVDs) are a leading cause of morbidity and mortality worldwide. Vascular endothelium plays a critical role in maintaining normal cardiovascular function by regulating vascular tone, thrombosis, and inflammation. Endothelial dysfunction, an early stage of atherosclerosis, is characterized by impaired vasodilatory capacity, pro-inflammatory and pro-thrombotic properties, and altered barrier function, which is a major predictor of cardiovascular events^1^. Another hallmark of endothelial dysfunction is the decreased bioavailability of nitric oxide (NOx), a critical signaling molecule synthesized by endothelial cells that regulate vascular tone and blood flow. Decreased production and bioavailability of NOx serve as key indicators of endothelial dysfunction^2^. This dysfunction can lead to a range of CVDs, such as diabetes, hypertension, atherosclerosis, coronary artery disease (CAD) and heart failure (HF)^3^.

Flow-mediated dilation (FMD) serves as a reliable indicator for evaluating endothelial function. It assesses the arteries’ capacity to respond to the release of endothelium-derived NOx during reactive hyperemia. This response is indicative of the endothelium’s integrity and its ability to regulate vascular tone, which is often compromised in endothelial dysfunction. Our previous studies have highlighted the importance of telomere length (TL), a biomarker of biological aging, in the pathogenesis of endothelial dysfunction^4^. Notably, leucocyte TL has also been reported as an independent risk factor for CVDs^5^. However, the role of cell-free plasma telomere length (cf-TL) in CVDs remains elusive. Most of the existing research on cf-TL has primarily focused on its implications in oncology^6,7^, leaving an unknown in the understanding of its potential impact on endothelial function and some CVDs, especially CAD and HF.

Machine learning (ML) techniques, with their ability to handle complex datasets and discover patterns that might be overlooked by traditional statistical methods, offer advantages in classification of diseases, predicting the onset and progression of diseases^8^. Compared to traditional logistic regression (LR) models, ML algorithms have demonstrated improved accuracy and predictive power, enabling more precise classification of diseases and better understanding of disease mechanisms. By leveraging advanced ML classifiers, researchers can more effective identification of key variables associated with cardiovascular events.

In this study, we aimed to examine the correlation between cf-TL, NOx, and FMD to explore its potential association with endothelial function, and to assess the potential of cf-TL as a biomarker in cardiovascular conditions, specifically in distinguishing between CAD and HF. Additionally, using ML models, we aimed to classify CAD and HF based on cf-TL and other key variables, and evaluate the relative contribution of cf-TL to the predictive accuracy of these models through SHAP analysis.

## Materials and methods

### Study participants

This was a retrospective review of data obtained from a previously established prospective protocol at the University Clinic Brandenburg^9^. The study included a total of 518 participants from the WalkByLab-database, which were enrolled between July 2019 and September 2022. The WalkByLab (www.walkbylab.com) is an ongoing trial that continuously screens for cardiovascular diseases to investigate, diagnose, and monitor cardiovascular risk and diseases in the underrepresented, non-metropolitan region of Brandenburg, Germany. All methods were performed in accordance with the relevant guidelines and regulations. A comprehensive description of the study design can be found in Zemmrich et al.^9^.

### Data collection and outcomes

Patient characteristics, including age, sex, body mass index (BMI), FMD, gender, diastolic blood pressure (DBP), systolic blood pressure (SBP), diabetes, hypertension, smoking, the current cardiovascular status, and laboratory parameters were prospectively collected. Laboratory parameters included hemoglobin (Hb), lipoprotein_a, total cholesterol, low density lipoprotein (LDL) cholesterol, high density lipoprotein (HDL) cholesterol, triglycerides, creatinine, estimated glomerular filtration rate (eGFR), and Glycated Hemoglobin A1c (HbA1c).

The primary outcome of interest was CAD. CAD was diagnosed based on a confirmed history of this condition, which included evidence of ≥ 50% coronary angiographic stenosis, or a previous record of any percutaneous coronary intervention or coronary artery bypass grafting. The secondary outcome of interest was HF. The current HF guidelines define three major categories including heart failure with reduced ejection fraction with left ventricular ejection fraction (LVEF) ≤ 40%, heart failure with preserved ejection fraction with LVEF ≥ 50%, and heart failure with mid-range ejection fraction with LVEF 41–49%^10^. In our study, HF was defined by the presence of a impaired pumping function of the heart. Specifically, this was defined by a left ventricular ejection fraction of less than 40% or a prior history of pacemaker implantation due to HF.

### Flow-mediated dilation (FMD) and nitric oxide (NOx)

FMD was measured using the AngioDefender™ system (Everist Health). The AngioDefender™ enables automatic and non-invasive measurement of brachial FMD. Based on the study by Heiss et al.^11^, which identified an FMD value of 6.5% as a critical cut-off for impaired endothelial function, we adopted FMD ≤ 6.5% as the classification threshold in our study.

To quantify plasma NOx levels, stable end products of nitrite and nitrate were measured [10] using a nitrate/nitrite colorimetric assay kit (Cayman Chemical). Nitrate was initially converted to nitrite by nitrate reductase, and then the Griess reagents were added. After incubation at room temperature for 10 min, the absorbance was measured at 550 nm using a microplate reader.

### Cell-free plasma Telomere length (cf-TL) measurement

5 ml peripheral blood was collected and centrifuged at 1000 g for 10 min. The resulting plasma was stored at -80 °C until use. Cell-free genomic DNA (cfDNA) was extracted from plasma using the QIAamp DNA Blood Mini Kit (Qiagen) and diluted to a final concentrations of 1 ng/µl for quantitative polymerase chain reaction (qPCR) amplification of cfDNA template. Each reaction contained 25 µl of gDNA, 2 µl of primer working solution, and 25 µl of PowerTrack™ SYBR Green Master Mix (Thermo Fisher Scientific). A two-step qPCR was performed with 60 cycles. All primers used in this study were synthesized by Eurofins Genomics Germany GmbH and their sequences are provided in Table S1. cf-TL was expressed as telomeric DNA (teloDNA) relative to acidic ribosomal phosphoprotein PO (36B4), which is a single copy gene and serves as internal reference. TL was calculated according to the formula: TL = 2^−ΔCT^, where ΔCT = CT_teloDNA_ − CT_36B4_.

### Machine learning (ML) modeling

The patient data included in 2022 year is used as the test dataset. The collected data of other years was randomly divided into training dataset and validation dataset with a ratio of 3 to 1. The training dataset was retained for subsequent analysis. The test dataset and validation dataset were strictly kept aside during model development and was used exclusively for internal validation to evaluate the final models. The significant associative factors used for ML modeling were screened by the least absolute shrinkage and selection operator (LASSO) regression and multivariate logistic regression (MLR). During ML modeling, we conducted a comprehensive search for optimal hyperparameter values within a reasonable range. Hyperparameter optimization strategies was random grid search^12^. After hyperparameter tuning, we applied nine algorithms on the training dataset to predict our interested outcomes.

The optimal threshold for binary classification was determined based on the Youden index. The nine algorithms used were decision tree (DT), random forest (RF), extreme gradient boosting (XGB), elastic net (ENet), support vector machine (SVM), multilayer perceptron (MLP), light gradient boosting machine (LGBM), K-nearest neighbor (KNN), and MLR. The ten-fold cross validation was carried during the training process of models.

Next, in the pursuit of enhancing the classification capabilities of ML models and models’ stability, we employed the stacking ensemble method to integrate several well-performing ML models into a single stack model. Stacking, also known as stacked generalization, is a powerful ensemble learning technique that combines the capability of multiple base models to improve overall performance and stability^13^. The stacking approach consists of two main steps. First, a set of diverse base ML models is trained on the training dataset. In our study, these refer to the nine different algorithmic ML models that we constructed. Then, the stacking model, is trained using the classifiers generated by the base models as input features. The stacking model is designed to learn the optimal combination of classifiers from the base models in order to generate a final classifier for the target variables, which leverages the strengths of individual models and mitigates their weaknesses, leading to a more robust and accurate classification^13^.

To provide insights into how features influence the outcomes, we employed the Shapley Additive Explanations (SHAP) approach^14^. SHAP is a model-agnostic method that aids in understanding the outcomes of a classifier or predictive model. The magnitude of SHAP values reflected the extent of each feature’s contribution to the model’s performance. This understanding can guide future research and potentially inform clinical decision-making.

### Statistics and software

All analyses were conducted in R and R Studio. Continuous variables were compared using the t test if data distribution met the criteria for normality. Otherwise, the Mann-Whitney test U test was used. *P* < 0.05 was considered statistically significant. The “mice” package in R was utilized to impute missing data. The LASSO and MLR analysis were carried out using R packages of “glmnet”. The ML part was carried out using R packages of “tidyverse”, “tidymodels”, “stacks”, and “fastshap”^13^.

## Results

### Participants characteristics

**Fig. 1:**
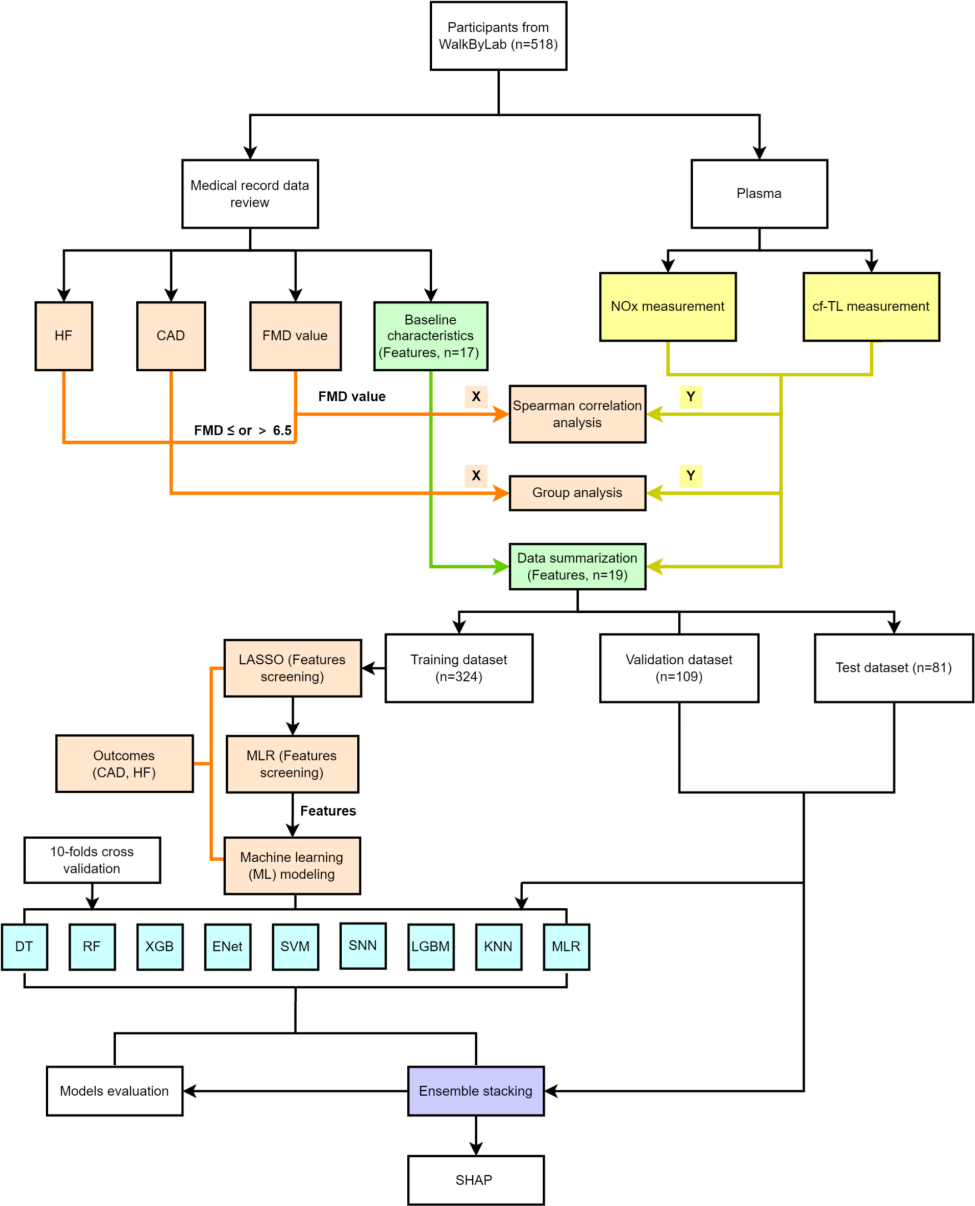
The flow chart of this study.

This study included a total of 518 participants, with a median age of 68 years and a median BMI of 27.15 kg/m2. There were 201 individuals had CAD and 164 individuals experienced HF. A total of 81 participants were included in the test dataset group, and the remaining 437 participants were randomly divided into a training dataset (*n* = 324) and a validation dataset (*n* = 109). Table 1 presents the demographic and baseline clinical characteristics of participants, comparing those with CAD, HF, and their respective control groups. The flow chart of this study is shown in Fig. 1.

**Table 1:** The demographic and baseline characteristics of participants. Continuous variables were reported as median [IQR]. Comparisons between groups using non-parametric Mann-Whitney test and chi-square test. p1 represents the p-value for the comparison between CAD and CAD-Controls. p2 represents the p-value for the comparison between HF and HF-Controls.

	Overall	CAD	CAD-Controls	p1	HF	HF-Controls	p2
n	514	201	313		164	350	
Age	68.50 [62.00, 76.00]	71.00 [64.00, 78.00]	68.00 [61.00, 75.00]	0.001	74.50 [68.00, 79.00]	66.00 [60.00, 73.00]	< 0.001
cf-TL	3.70 [2.64, 5.11]	3.68 [2.81, 5.32]	3.72 [2.49, 5.00]	0.346	3.83 [2.98, 5.40]	3.65 [2.43, 5.01]	0.014
NOx	2.95 [2.55, 3.32]	2.95 [2.55, 3.36]	2.95 [2.56, 3.23]	0.751	3.05 [2.63, 3.44]	2.90 [2.50, 3.21]	0.002
BMI	27.15 [24.50, 30.10]	27.90 [25.50, 31.40]	26.50 [24.10, 29.70]	< 0.001	27.70 [25.40, 31.02]	26.85 [24.10, 30.00]	0.01
eGFR	79.00 [63.85, 91.47]	72.50 [55.70, 87.60]	82.81 [69.10, 92.00]	< 0.001	66.90 [52.30, 80.00]	84.15 [71.00, 92.88]	< 0.001
Hb	138.00 [130.00, 145.00]	138.00 [128.00, 145.00]	138.00 [130.00, 145.00]	0.741	134.00 [125.00, 141.00]	139.00 [132.00, 146.00]	< 0.001
HbA1C	5.75 [5.59, 6.10]	5.84 [5.50, 6.50]	5.72 [5.60, 5.98]	0.02	5.90 [5.60, 6.43]	5.70 [5.58, 6.00]	0.001
HDL_cholesterol	1.41 [1.17, 1.65]	1.33 [1.09, 1.55]	1.47 [1.22, 1.77]	< 0.001	1.38 [1.18, 1.57]	1.44 [1.17, 1.72]	0.036
Creatinine	78.16 [68.00, 92.00]	86.00 [76.00, 102.00]	74.00 [65.00, 85.00]	< 0.001	89.50 [75.00, 110.00]	74.16 [66.00, 85.00]	< 0.001
LDL_cholesterol	2.68 [1.93, 3.51]	2.08 [1.60, 2.64]	3.10 [2.48, 3.88]	< 0.001	2.20 [1.65, 2.88]	2.93 [2.21, 3.70]	< 0.001
Lipoprotein_a	7.29 [3.00, 39.50]	12.00 [3.79, 62.60]	6.17 [3.00, 24.41]	< 0.001	10.04 [3.25, 61.35]	6.54 [3.00, 31.22]	0.089
Total_cholesterol	4.56 [3.72, 5.45]	3.79 [3.23, 4.54]	5.09 [4.29, 5.79]	< 0.001	3.94 [3.37, 4.79]	4.89 [4.00, 5.63]	< 0.001
Triglycerides	1.23 [0.89, 1.83]	1.32 [0.96, 1.89]	1.20 [0.86, 1.75]	0.135	1.33 [0.96, 1.93]	1.19 [0.88, 1.78]	0.218
DBP	79.00 [71.00, 85.00]	76.00 [69.00, 84.00]	80.00 [73.00, 87.00]	< 0.001	76.69 [69.00, 84.00]	80.00 [73.00, 86.00]	0.004
SBP	139.00 [128.00, 150.00]	135.00 [125.00, 146.00]	141.00 [130.00, 152.00]	0.007	140.00 [127.00, 150.50]	138.00 [128.00, 149.00]	0.605
Male (%)	278 (54.09)	140 (69.65)	138 (44.09)	< 0.001	104 (63.41)	174 (49.71)	0.005
Smoking (%)	222 (43.19)	105 (52.24)	117 (37.38)	0.001	73 (44.51)	149 (42.57)	0.75
Diabetes (%)	104 (20.23)	55 (27.36)	49 (15.65)	0.002	47 (28.66)	57 (16.29)	0.002
Hypertension (%)	371 (72.18)	184 (91.54)	187 (59.74)	< 0.001	153 (93.29)	218 (62.29)	< 0.001

### cf-TL correlated with NOx and FMD

**Fig. 2:**
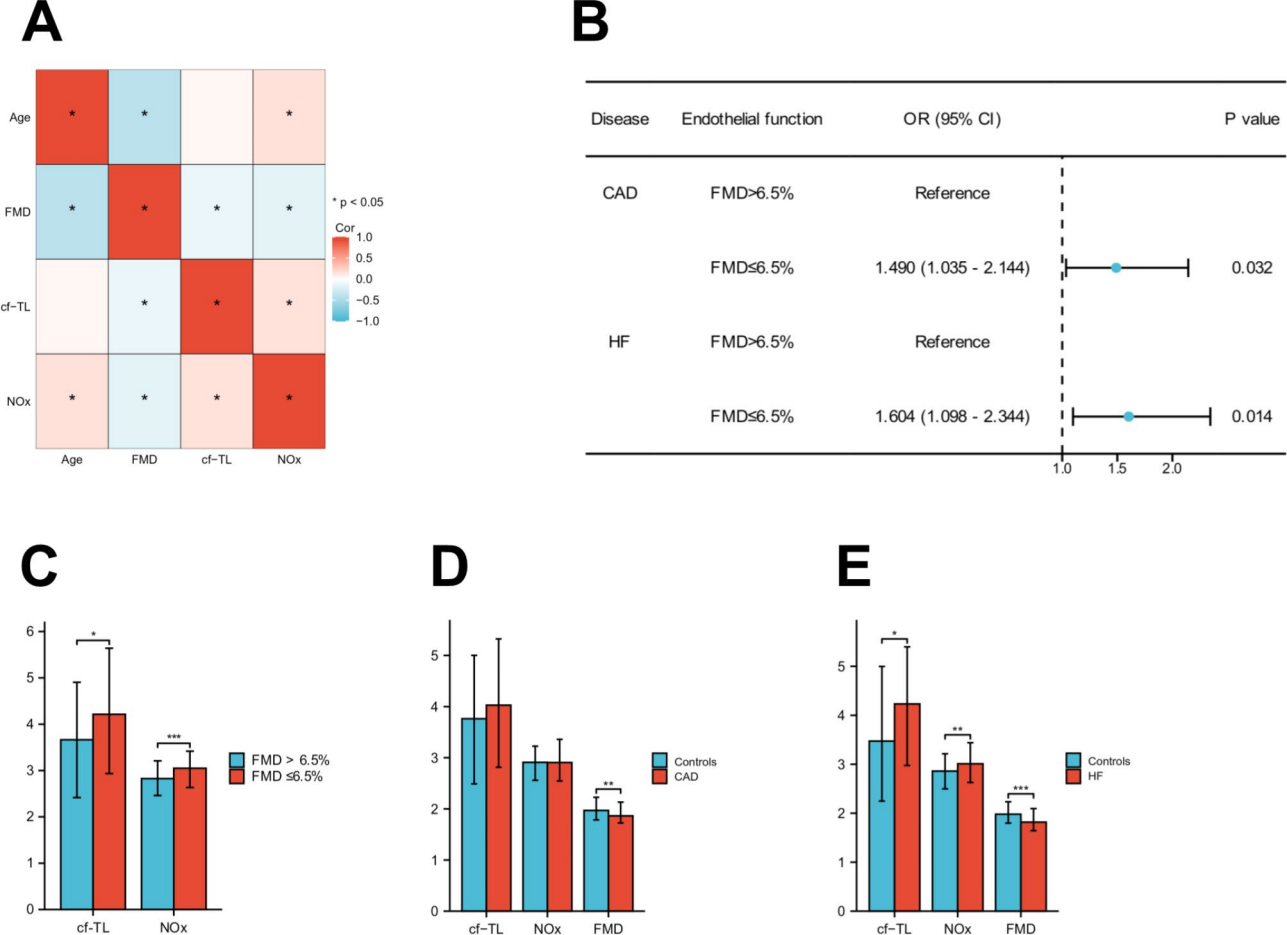
(**A**) Spearman correlation analysis of age, FMD, cf-TL, and NOx. (**B**) Forest plot for the relationship between FMD ≤ 6.5% and CAD as well as HF. (**C**-**E**) The levels of cf-TL and NOx in participants with FMD ≤ 6.5%, CAD, and HF, respectively.

Spearman correlation analysis showed that age, FMD, cf-TL, and plasma NOx were correlated with each other, as depicted in Fig. 2A. Specifically, cf-TL was correlated with plasma NOx (correlation coefficients, Cor = 0.158) and FMD (Cor = -0.128) (*P* < 0.001).

### FMD ≤ 6.5% was a crucial factor in the development of CAD and HF

In Heiss’s study^11^, the author suggested FMD value of 6.5% as a cut-off for the impairment of endothelial function. Based on this, we explored the relationship between FMD ≤ 6.5% and CAD, as well as HF. Univariate analyses indicated that FMD ≤ 6.5% was significantly associated with an increased risk of CAD (Odds Ratio, OR: 1.490, 95% confidence interval, CI: 1.035–2.144, *P* = 0.032) and HF (OR: 1.604, 95% CI: 1.098–2.344, *P* = 0.014) (Fig. 2B).

### Longer cf-TL correlated with FMD ≤ 6.5% and HF

The study found that participants with FMD ≤ 6.5% showed significantly longer cf-TL (*P* = 0.029) and higher NOx level (*P* < 0.001) (Fig. 2C; the values were natural log transformed). However, there was no significant association between cf-TL and CAD (*P* > 0.05) (Fig. 2D). In the HF group, participants had longer cf-TL (*P* = 0.034) and higher levels of NOx (*P* = 0.002), and a significant decreased in FMD (*P* < 0.001) (Fig. 2E).

### cf-TL was an significant variable for CAD and HF

**Fig. 3:**
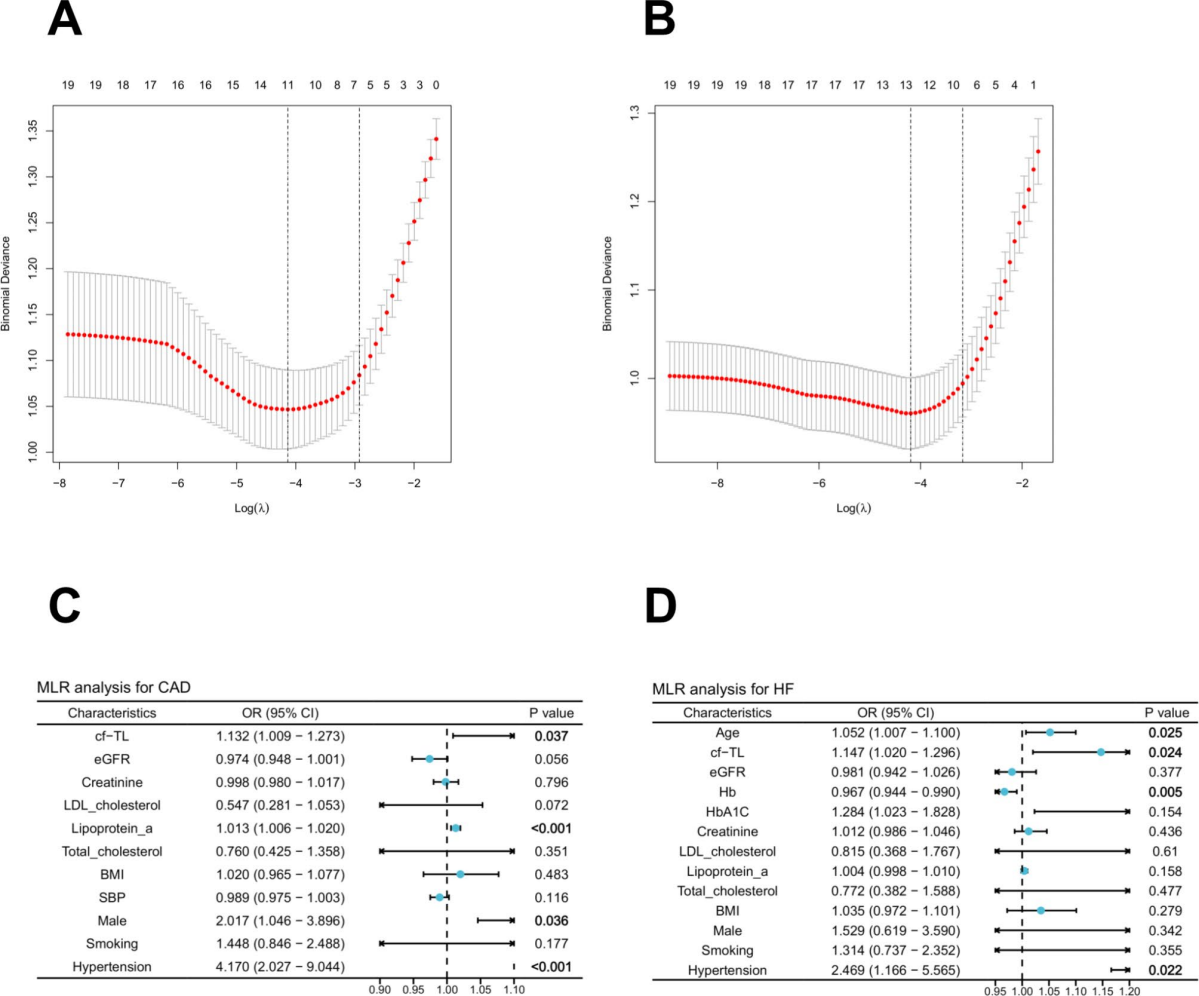
(**A**-**B**) LASSO analysis for CAD, and HF. (**C**-**D**) The results of MLR analysis for CAD and HF.

In our study, a total of 19 features were included. To reduce the dimensionality of the data and select the optimal predictive features, LASSO regression was performed to determine the nonzero coefficient variables in CAD and HF, respectively. Following LASSO, the variables were reduced to 11 potential variables in CAD, and 13 potential variables in HF (Fig. 3A-B). To further control the influence of confounding features, the above variables were analyzed by MLR. The results (Fig. 3C-D) indicated that for CAD, the significant variables included cf-TL, lipoprotein_a, gender (male), and hypertension (*P* < 0.05). Additionally, age, cf-TL, Hb, and hypertension were identified as significant variables for HF (*P* < 0.05).

Good ML classification models often emphasize the use of fewer variables. This is because fewer variables can reduce model complexity and decrease the risk of overfitting. Moreover, using fewer variables can also enhance the interpretability of the model, and reduce the cost associated with data collection and processing. Therefore, we chose these four significant variables (cf-TL, lipoprotein_a, male, and hypertension; *P* < 0.05) to construct ML models for classification of CAD. Similarly, for the classification of HF, we incorporated four key features into our models: age, cf-TL, Hb, and hypertension (*P* < 0.05).

## ML classifiers

### Base ML modeling

**Fig. 4:**
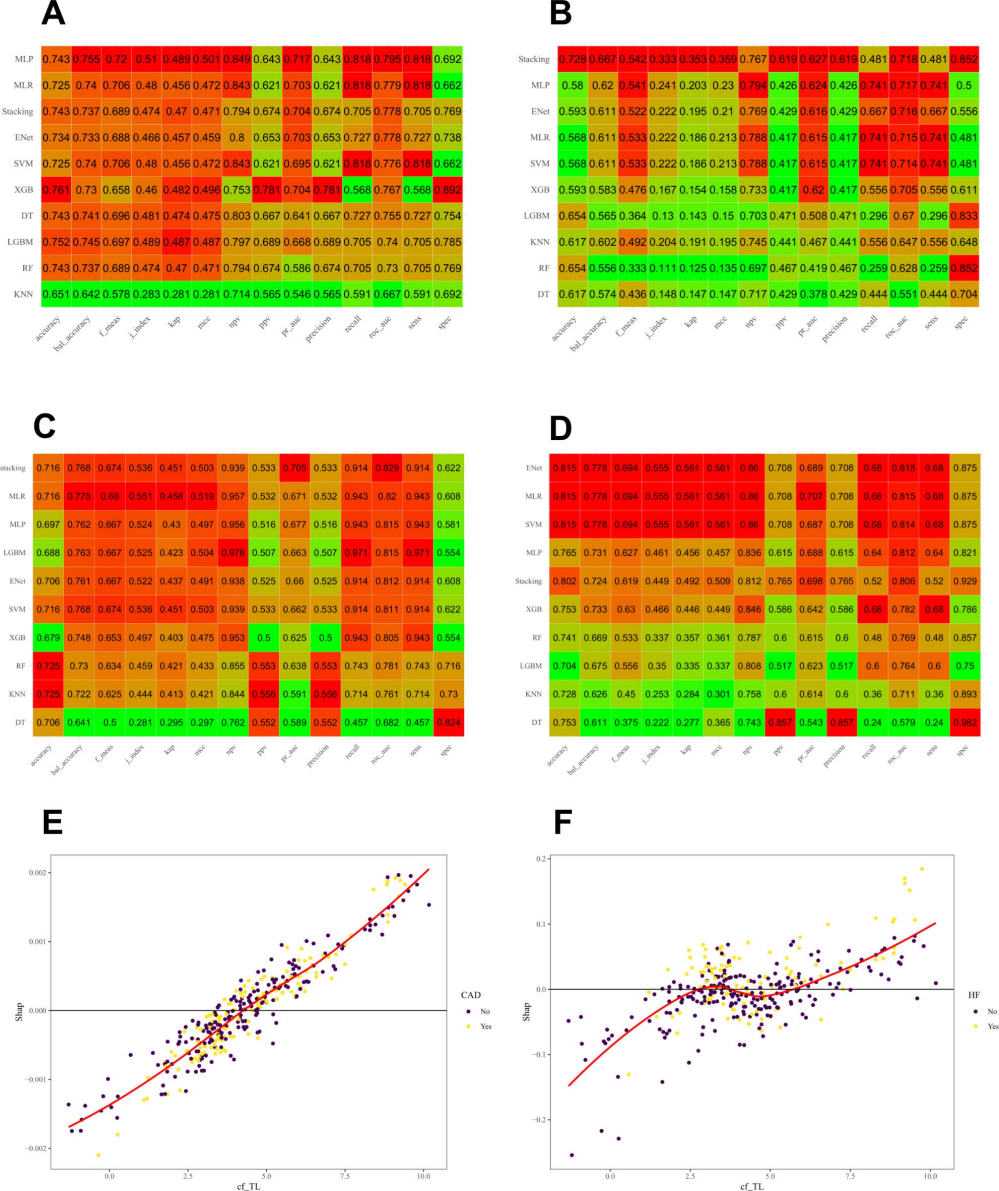
(**A**-**B**) The evaluation metrics for CAD models on the validation dataset and test dataset. (**C**-**D**) The evaluation metrics for HF models on the validation dataset and test dataset. (**E**-**F**) The dependence plots of SHAP for cf-TL on the classification of CAD and HF.

A total of 18 ML models were constructed using nine ML algorithms for CAD and HF classification. The performance of the models for CAD has been comprehensively evaluated through the evaluation metrics summarized in Fig. 4A and B for the validation dataset and test dataset, respectively. Similarly, Fig. 4C and D present a detailed assessment of the models’ performance for HF in the validation dataset and test dataset, respectively.

### Stacking ML modeling

Moreover, to further potentially improve the performance and stability of our models, we trained these base ML models and generate an ensemble stack model, using LASSO with 20 distinct penalty values. This approach allowed us to impose the strengths of multiple models and enhance the overall classification capabilities and stability. During the ensemble stacking process, LASSO was used as the meta-model, while several high-performing models were employed as base models for each outcome. Specifically, we have selected the top four models with the best performance as the base models. Specifically, SVM, MLP, MLR, and ENet models were used as base models for the classification of CAD For the classification of HF, the ENet, LGBM, MLP, and MLR models were used as base models.

### Optimal classifier model

The evaluation metrics in Fig. 4A - B indicated that in CAD classifiers, the MLP model had the highest AUC of 0.795 on the validation dataset, which also demonstrated a good performance on the test dataset with an AUC of 0.717. We considered the MLP model as the optimal model for CAD.

In HF classifiers (Fig. 4C - D), the stacking model had the highest AUC value of 0.829 on the validation dataset, translated favorably to the test dataset where it achieved an AUC of 0.806. We considered the stacking model as the optimal model for HF.

### Feature interpretation

To visually explain how cf-TL influences the outcomes in our models, we used SHAP approach (Fig. 4E-F). The y-axis values represent the SHAP values, which quantify the impact of each feature on the model prediction, while the x-axis values represent the feature values themselves.

In the cf-TL dependent plot for CAD illustrated in Fig. 4E, the SHAP values ranged from approximately − 0.002 to 0.002, indicating that the contribution of cf-TL to the model’s output is minimal. This suggests that cf-TL may not be a significant determinant in assessing CAD risk. Conversely, in the cf-TL dependent plot for HF shown in Fig. 4F, the SHAP values exhibited a wider range from − 0.3 to 0.2. This greater variability in SHAP values suggests that cf-TL has a more pronounced impact on the predictive outcomes of the HF model. In summary, cf-TL’s influence on CAD is limited, while its predictive contribution to HF is substantial.

## Discussion

Cell-free DNA (cf-DNA) is the fragmented double-strand DNA released from dying cells into circulating blood, serving as a marker of cell death. Previous studies have demonstrated a positive correlation between plasma cf-DNA levels and prognostic mortality in stroke patients, highlighting the potential of cf-DNA as a biomarker for severe cardiovascular events^15^. Building on this, our study explored the role of cf-TL, a specific subset of cf-DNA, in cardiovascular conditions, specifically CAD and HF. Normally, longer leucocyte TL may indicate a physiological condition or a compensation stage, reflecting healthy cellular maintenance. In contrast, longer cf-TL may indicate a pathological condition or a decompensation stage, indicative of ongoing cellular stress and damage^16,17^. The shared pathophysiological basis of stroke and CAD, namely atherosclerosis, suggested that there would be potential significance of investigating the role of cf-TL in cardiovascular conditions.

In the present study, we unveiled a significant association between prolonged cf-TL and a decreased FMD value which is an indicator of endothelial function. Specifically, FMD ≤ 6.5% stands out as a pivotal factor in the development of CAD and HF. The observed correlation with lower FMD implies that longer cf-TL reflected a more profound degree of endothelial dysfunction, which is a harbinger of atherosclerosis and subsequent cardiovascular events. Furthermore, the co-correlation between cf-TL, NOx levels, and FMD values strengthened the hypothesis that endothelial dysfunction lies at the heart of these associations. Endothelial dysfunction disrupts vasodilation mechanisms, exacerbates oxidative stress, and fosters inflammation, collectively paving the way for the initiation and progression of atherosclerosis, CAD, and HF. This comprehensive understanding underscored the pivotal role of endothelial health in maintaining cardiovascular well-being and highlighted the potential of cf-TL as a prognostic tool in this context.

Therefore, we speculate that cf-TL could serve not only as a marker of endothelial health but also as an indicator of broader cardiovascular risk. Despite this potential, our study did not find a significant association between cf-TL and CAD. However, in the HF group, participants demonstrated longer cf-TL, suggesting a possible link between cf-TL and HF. Next, we employed LASSO and MLR to identify significant associative factors for CAD, and HF. We identified cf-TL, lipoprotein_a, gender (male), and hypertension as significant associative factors for CAD (*P* < 0.05). Age, cf-TL, Hb, and hypertension were identified as significant associative factors for HF (*P* < 0.05).

Among these significant associative factors, the link between lipoprotein_a and CAD has been extensively researched; Lipoprotein_a is a type of lipoprotein particle that has been linked to atherosclerosis development and progression^18^. Its level have been demonstrated to contribute to the formation of arterial plaques and raise the risk of CAD^18,19^. Male gender has long been known as a non-modifiable risk factor for CAD. Men have a higher prevalence of CAD than women, and the causes of this gender gap are multifaceted and may include hormonal, genetic, and lifestyle variables^20^.

Our study also highlights age and Hb levels as significant associative factors in HF. Age-related changes in cardiac structure and function, along with chronic low-grade comorbidities superimposed on the limited cardiac regeneration capacity, contribute to the increased risk of HF in older individuals^21^. Hb is responsible for transporting oxygen to body tissues, and its levels reflect the oxygen-carrying capacity of the blood. Reduced Hb levels may lead to inadequate tissue oxygenation, resulting in cardiac dysfunction and the progression of HF. But anemia in HF remains an enigma. The discussion regarding anemia as a marker of severe disease or the cause of poor outcomes in HF patients is still ongoing^22^. Thus, monitoring and optimizing Hb levels could be a potential therapeutic approach to mitigate the risk of HF and improve patient outcomes.

Hypertension, a well-known risk factor for CVDs, was identified as an significant associative factor in CAD and HF. Indeed, elevated blood pressure contributes to oxidative stress, and vascular remodeling, ultimately leading to the development of CVDs. These aligned with previous research findings. Effective management and control of hypertension are critical in reducing the burden of CAD and HF.

Finally, cf-TL was identified as an significant associative factor for CAD and HF in our study. The longer the cf-TL, the higher the risk of CAD and HF. By extending our understanding of cf-TL’s role in disease progression, we can better reveal its significance as a significant associative factor of CAD and HF.

Another aim of this study was to use ML techniques to construct classifiers for the classification of CAD and HF. The application of ML methods in disease diagnosis or prediction has emerged as a rapidly evolving field. However, the complexity and interpretability of these ML models can sometimes be challenging. In our study, we aimed to get a balance between model performance and simplicity by incorporating only the identified significant associative factors in our ML models. By using the associative factors that were determined by LASSO and MLR analyses, we were able to reduce the dimensionality of the dataset and focus on the most relevant factors associated with the outcomes of interest. This approach not only facilitated the development of simplified models but also enhanced the interpretability of the results. Moreover, it also has the potential to reduce the risk of overfitting, which occurs when the model captures noise or irrelevant patterns in the data^23^. By excluding less relevant variables, we can mitigate the potential for overfitting and improve the generalizability of the models to unseen data^24^. Additionally, model simplicity enhances the clinical applicability. Complex models with numerous features may be challenging to implement in real-world clinical settings. By using a reduced set of important variables, our models become more user-friendly and easier to translate into practical clinical tools for risk assessment and decision-making.

The present study showed that the ML models demonstrated superior performance than traditional MLR model in classifing each outcome, respectively. In order to further enhance the predictive performance and stability of our models, we employed a stacking approach on top of the base ML models. Stacking is a powerful ensemble technique that combines multiple individual models to create a more stable and accurate model^13^. By leveraging the diversity and complementary strengths of different base models, stacking allows us to capture a broader range of patterns and improve the overall classification performance and stability.

In this study, the MLP and stacking models demonstrated outstanding performance in classifying CAD and HF, respectively. These models achieved the highest AUC on the validation dataset, indicating superior classification ability, and continued to perform well on the independent test dataset. However, based on the SHAP analysis, cf-TL appears to have a limited role in classifying CAD, as its contribution to the model is marginal. Considering that no significant elevation of cf-TL was observed among participants in the CAD group. This lack of association indicates that cf-TL may have limited relevance in CAD classification, which aligns with the minimal impact observed in the SHAP value analysis.

In contrast, for HF classification, the SHAP values displayed a wider range, from − 0.3 to 0.2, suggesting a more substantial contribution of cf-TL to the model of HF classification. Moreover, a significant elevation of cf-TL was observed among HF participants during our comparative analysis. This association reinforces the idea that cf-TL plays a more prominent role in HF risk assessment compared to CAD. In conclusion, these findings suggest that cf-TL may serve as a critical predictor for HF, contributing to improved predictive performance in the HF model.

The interpretability of ML models is essential for translating complex classification outcomes into actionable insights. In this study, SHAP analysis was utilized to provide a deeper understanding of the contribution of individual variables, such as cf-TL, to the predictive models for CAD and HF. The explanation serves two main purposes: first, it enhances the transparency of the model’s decision-making process by identifying the variables most influential in predicting disease outcomes. This builds trust in the model’s predictions and enables clinicians to assess whether the identified risk factors align with known pathophysiological mechanisms, thereby validating the model’s clinical relevance.

To further ensure the robustness and generalizability of our findings, we implemented a temporal data split, holding out the most recent year’s data as the test set while using earlier data for training and validation. This approach closely mimics real-world clinical settings, where new patient data must be accurately predicted based on historical data. By separating the test set temporally, we aimed to prevent data leakage, ensuring that the model captures only the relationships relevant to the research question and not confounding patterns unrelated to future patient outcomes. The results from this temporal split showed consistent performance in both CAD and HF classification, with AUC values reflecting the model’s ability to generalize to new, unseen data.

Additionally, the SHAP analysis, combined with the temporal data split, highlighted cf-TL’s minimal impact on the CAD model but significant influence on the HF model. Since cf-TL exhibited a broader range of SHAP values in the HF prediction model, this suggests a more prominent role in heart failure pathophysiology, potentially linked to cellular aging and endothelial dysfunction. These insights guide future clinical investigations, such as exploring cf-TL’s role in monitoring HF progression or developing telomere-based therapeutic strategies. Moreover, temporal data split and SHAP explanations assist in identifying patient-specific risks, offering clinicians additional perspectives for patient risk assessment. However, it is important to note that SHAP, as with any other noncausal explanation method, should not be solely relied upon for guiding clinical decision making due to its inability to establish causality^25^. Instead, it should be regarded as a complementary tool within the broader context of clinical information.

Our study has several limitations. First, this study was a retrospective study of data from a prospective study. We established classification models based on retrospective data, while accurate prediction of the risk of these outcomes depends on long-term prospective cohort studies. Second, we only conducted internal validation, and did not conduct an external validation because we did not have another independent population. In our future research, we will complete the collection of cohort data to verify the results of this study.

In summary, our research has demonstrated a correlation between longer cf-TL and declined endothelial function, and highlighted the differential role of cf-TL in patients with HF and CAD. The prolonged cf-TL observed in HF patients, in contrast to the non-significant differences in CAD patients, suggest that cf-TL may serve as a more relevant biomarker in the context of HF. Additionally, ML models demonstrated stronger predictive performance for HF classification compared to CAD, with cf-TL contributing more significantly to the HF model’s predictions, as indicated by the broader range of SHAP values. These findings underscore the potential of cf-TL as a valuable predictor for HF, providing a foundation for future studies to explore its mechanistic role and clinical utility in cardiovascular disease management. Further research with larger cohorts is warranted to validate cf-TL’s diagnostic and prognostic capabilities across different cardiovascular conditions.

## Electronic supplementary material

Below is the link to the electronic supplementary material.

Supplementary Material 1

## Data Availability

The data for this study is from our WalkByLab-database (www.walkbylab.com), which is not publicly accessible. The R code is available by contacting the Corresponding author.
